# Trend of malaria cases in Kaduna State using routine surveillance data, 2011-2015

**DOI:** 10.11604/pamj.supp.2019.32.1.13735

**Published:** 2019-01-24

**Authors:** Ummulkhulthum Abubakar Bajoga, Hadiza Sabuwa Balarabe, Abayomi Akintunde Olufemi, Mahmood Muazu Dalhat, Ibrahim Baffa Sule, Muhammed Sani Ibrahim, Ayo Stephen Adebowale, Babatunde Olubayo Adedokun, Mohammed Yahaya, Ike Oluwapo Oyeneye Ajayi, Patrick Mboya Nguku, Olufemi Olamide Ajumobi

**Affiliations:** 1 Nigeria Field Epidemiology and Laboratory Program, Abuja, Nigeria; 2 Kaduna State Primary Health Care Development Agency, Nigeria; 3 Department of Community Medicine, Ahmadu Bello University Zaria, Nigeria; 4 Department of Epidemiology and Medical Statistics, University of Ibadan, Nigeria; 5 Department of Medical Microbiology and Parsitology, College of Health Sciences, Usman Danfodio University, Sokoto, Nigeria; 6 African Field Epidemiology Network, Nigeria Country Office, Nigeria

**Keywords:** Malaria, surveillance, Kaduna, Nigeria, trend

## Abstract

**Introduction:**

in 2015, 212 million malaria cases and 429,000 malaria deaths were estimated globally. Kaduna State, located in northern Nigeria had a malaria prevalence rate of 36.7% among children less than 5 years old which was higher than the national average of 27%. We assessed the trend of malaria cases in Kaduna State from 2011 to 2015, to analyse trend of malaria in Kaduna as well as describe malaria in time, place and person.

**Methods:**

we conducted secondary data analysis of Kaduna State malaria data between January 2011 and December 2015. Data were extracted from the Integrated Disease Surveillance and Response (IDSR) 003 form. Data of uncomplicated malaria defined as “any person with fever or history of fever within 24 hours; without signs of severe disease (vital organ dysfunction)” was analysed. In IDSR, a case of malaria is based on presumed diagnosis. Frequencies and proportions were calculated. We also conducted trend analysis of incidence of malaria.

**Results:**

in the period under study, 1,031,603 malaria cases were recorded with 238 deaths (CFR = 0.23 per 1,000). There was a downward trend with a slope of -3287.2. The data showed higher seasonal variation for quarters 2 (1430.96) and 3 (Q2 = 6,460.23) compared to Quarters 1 (6,857.19) and 4 (-1,034.01). Overall, the age group 12 -59 months had the highest number of incident cases 225, 537 (20.3%). Malaria death was highest in children 1 to 11 months (26.5%) and least, in children 0 -28 days (2.5%). CFR was also highest in children 1 to 11 months (0.45 per 1,000). The highest incidence of malaria cases was in Jaba Local Government Area (47.7%) and the least, in Lere (2.4%).

**Conclusion:**

there was a decreased incidence of malaria from 2011 to 2015. Malaria was most common in the second and third quarters of each year. Age group 12-59 months was most affected. Kaduna State Malaria Programme should sustain the programs it is implementing and focus more on the under-five years age group.

## Introduction

In 2015, 212 million malaria cases and 429,000 malaria deaths were estimated globally [1]. About 90% of the cases and 92% of the deaths were in World Health Organisation (WHO) African region [1]. Special groups like children under 5 and pregnant women are susceptible to malaria. Malaria transmission depends on factors related to vector, parasite, human host and environment. Environmental factors include climatic conditions such as rainfall, humidity and temperature patterns [2]. Although malaria incidence is estimated to have decreased by 41% globally between 2000 and 2015, 91 countries including Nigeria have ongoing malaria transmission [1]. The proportion of malaria accruable to Nigeria is 26%. The case fatality rate gives an indication of the severity of the disease [3]. Nigeria accounts for up to 29% of malaria disease burden in Africa [1]. Furthermore, 60% of outpatient visits and 30% of hospitalisations among children under 5 are due to malaria [4]. According to Nigeria Malaria Indicator Survey (MIS), malaria parasite prevalence among children under-five years is 27.4% [5]. National Malaria Elimination Programme coordinates all malaria activities in Nigeria with oversight from the Federal Ministry of Health [6]. The goal of the National Malaria Strategic Plan 2014 -2020 is to reduce malaria burden to pre-elimination level and malaria mortality to zero. Assessing malaria surveillance data is important in monitoring progress towards elimination [7]. Kaduna State, Nigeria was noted to have prevalence of malaria of 36.7% in children under 5 which was higher than the national average of 27% [5]. However, high ownership of long lasting insecticidal nets (LLIN) of 91.6% and use of 66.1% [5] have also been documented in the Malaria Indicator Survey (MIS) 2015. There is a need to design new and appropriate control strategies apart from what is already established based on evidence of malaria yearly incidence trends. Although studies have been conducted in different fields like salmonellosis, rubella and cancer using surveillance data only few studies were done using malaria surveillance data [8-11]. We conducted the study to assess the trend of incidence of malaria in Kaduna State from 2011 to 2015, to identify relationship between socio-demographic characteristics and malaria burden.

## Methods

### Study area

Kaduna State is in the Northwest geopolitical zone of Nigeria and is divided into 23 Local Government Areas (LGAs) and lies 10°20’N 7°45’E. Kaduna had a population of 6,113,5503 in 2006 [12] that comprised of 50.6% males. The projected populations of each of the years under study were 7,032,808; 7,243,792; 7,461,106; 7,684,939 and 7,915,487 in the five years under study respectively with a growth rate of 2.5%. Temperature ranges from 15.9 to 35.4°C and altitude of 612m. Rainfall varies from 0.0 to 825.0 mm/month [13]. The rainy season starts from March and peaks in August, then declines till October. The high-transmission season for malaria is between June and October, whereas low transmission is between November and May [14]. The first LLIN mass campaign in Kaduna state was in 2010. LLIN were distributed free of charge to pregnant women in the State during the Maternal, Newborn and Child Health Week (MNCHW) in 2014. In March 2016, there was also state wide LLIN mass campaign.

### Study design

**Figure 1:**
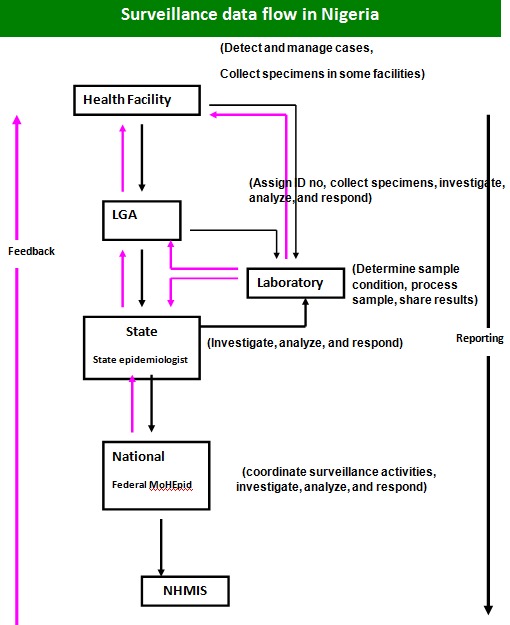
flow of information in IDSR

We conducted secondary data analysis of malaria data of January 2011 to December 2015. The data were extracted from the Integrated Disease Surveillance and Response (IDSR) 003 form. The IDSR 003 tool collects data monthly on 40 priority health events including malaria. The data is collected from health facilities and sent to LGA Disease Surveillance and Notification Officer (DSNO). The officer collates the data from all the facilities in his/her Local Government and transmits the data to the state DSNO who in turn transmit this to the Federal Ministry of Health monthly. Samples collected are sent to the laboratory where they are analysed and results sent to the health facility, LGA or State. Feedback is obtained through the same channels (Figure 1).

### Case definition

According to the IDSR guidelines, uncomplicated malaria is defined as “any person with fever or history of fever within 24 hours; without signs of severe disease (vital organ dysfunction)” [15].

### Data management

We conducted data analysis with Microsoft Excel and SPSS. Frequencies and proportions of malaria cases were calculated. We plotted the pattern of malaria to establish seasonality in the reported number of cases from 2011 to 2015. The additive moving average time series model of the form

$$y_{t}=T_{L_{t}}+SV_{L_{t}}+\in_{t}$$

was obtained from the plot based on the displayed pattern with the assumption of homogeneity in variance, independence and normality. A three-month period moving average was estimated for yt and the trend line was determined thereafter using iterative equation;

$$T_{L_{t}}=\frac{y_{t-1}+y_{t}+y_{t-1}}{3}$$

based on the stated assumptions, εt = 0. Therefore,

$$SV_{L_{t}}=y_{t}-TL_{t}$$

To de-seasonalise the computed seasonal variation, the seasonal variation adjusted factors were obtained using a dummy variable. The seasonality referred to here was the four quarters of the year. To model the seasonal pattern, the seasonal variation factor, SVt, was de-seasonalised using a dummy variable which ensures that a seasonal parameter ß for each season j was added to the trend in each appropriate time period. The seasonal parameter, the increase in trend line per quarter and the estimated trend were used to predict cases of malaria in each quarter for year 2016.

$$y_{2016}=TL_{t}+{\text{β}}_{ij}+\text{ITL per Qtr}$$

Incidence of malaria using population figures projected from 2006 census figures as denominators, was calculated. Completeness of surveillance data from each LGA was determined. The independent variable was malaria cases and the dependent variables were LGA, month, year, age groups, inpatient, outpatient cases and death.

### Ethical consideration

This is a secondary data analysis of routinely collected data. Permission was sought from Kaduna State Primary Health Care Development Agency for the use of the data. The data did not include personal identifiers.

## Results

Malaria accounted for 64.8% of all outpatient cases seen in Kaduna State over the 5 years. In the period under study, 1,031,603 malaria cases were recorded with 238 deaths (CFR = 0.23 per 1,000). Incidence of malaria showed a steady decrease (apart from in 2013). Incidence in 2011 was 4.56% and 1.02% in 2015.The case fatality rate fluctuated between 2011 and 2015, with no sign of decline (Table 1). Table 2 shows trend, seasonal variation and 2016 predicted number of reported cases of malaria in Kaduna state.

$$\text{Increase in Trend line per quarter (ITL per Qtr)=}\frac{T_{L}{}_{{}_{n-1}}-T_{L_{2}}}{n-1}=-3288.55$$

**Table 1:** malaria incidence and case fatality rates, Kaduna State, 2011-2015

Year	Population	Malaria cases	Incidence %	Death	CFR per 1,000
2011	7,032,808	320,475	4.56	59	0.18
2012	7,243,792	211,974	2.93	62	0.29
2013	7,461,106	211,416	2.83	47	0.22
2014	7,684,939	207,368	2.70	49	0.24
2015	7,915,487	80,370	1.02	21	0.26
**Total**		1,031,603		238	

**Table 2:** trend, seasonal variation and 2016 predicted number of reported malaria cases Kaduna State, 2011-2015

Year	Quarter	Number of reported cases of malaria	3-Qtr Moving Total	Trend	Seasonal Variation	Predicted value 2016
**2011**	Q_1_	70,002	-	-	-	
	Q_2_	72,452	228,154	76,051.33	-3,599.33	
	Q_3_	85,700	250,473	83,491	2,209	
	Q_4_	92,321	241,465	80,488.33	11,832.67	
**2012**	Q_1_	63,444	221,051	73,683.67	-10,239.7	
	Q_2_	65,286	194,337	64,779	507	
	Q_3_	65,607	148,530	49,510	16,097	
	Q_4_	17,637	125,061	41,687	-24,050	
**2013**	Q_1_	41,817	106,006	35,335.33	6,481.667	
	Q_2_	46,552	149,121	49,707	-3,155	
	Q_3_	60,752	169,599	56,533	4,219	
	Q_4_	62,295	162,722	54,240.67	8,054.333	
**2014**	Q_1_	39,675	165,444	55,148	-15,473	
	Q_2_	63,474	166,926	55,642	7,832	
	Q_3_	63,777	167,693	55,897.67	7,879.333	
	Q_4_	40,442	124,151	41,383.67	-9,41.667	
**2015**	Q_1_	19,932	87,295	29,098.33	-9,166.33	
	Q_2_	26,921	67,685	22,561.67	4,359.333	
	Q_3_	20,832	60,438	20,146	686	
	Q_4_	12,685	-	-	-	15,823.44
**2016**	Q_1_					6,711.71
	Q_2_					11,711.31
	Q_3_					13,452.03
	Q_4_					2,669.24

**Figure 2:**
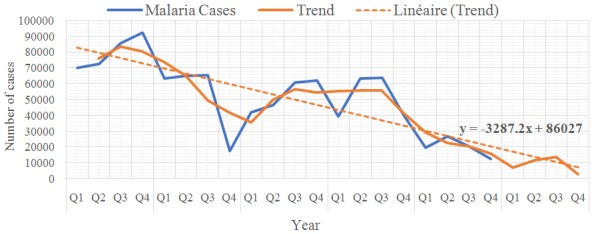
observed values, trend and trend line of the number of reported cases of malaria in Kaduna State

**Figure 3:**
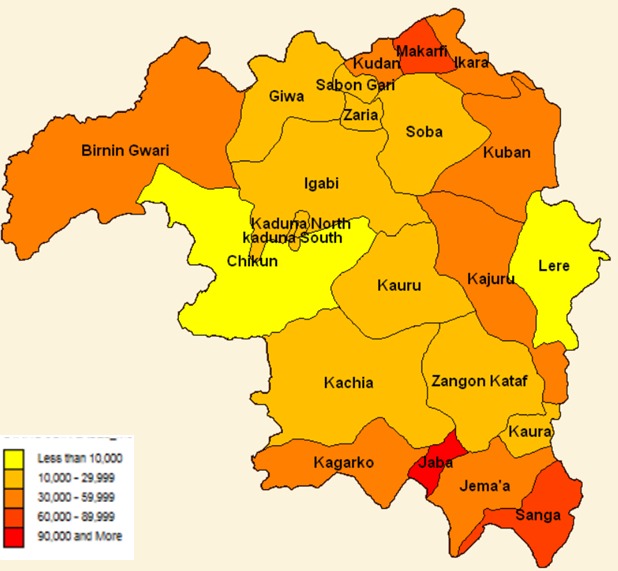
distribution of malaria cases according to LGA, 2011-2015

**Figure 4:**
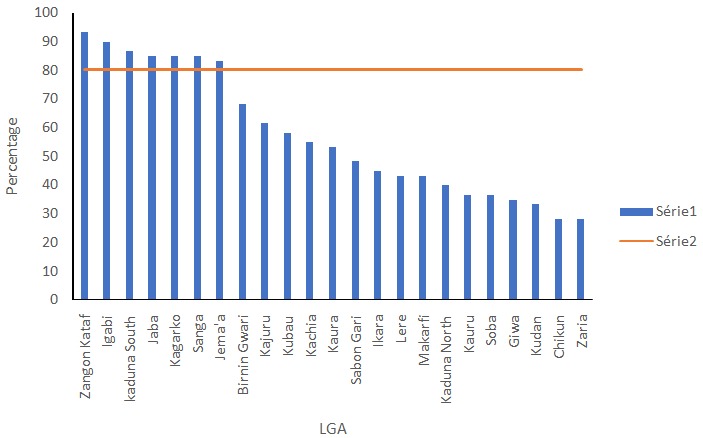
bar chart showing percentage of complete surveillance reports per LGA

In the years under review, the data showed higher seasonal variation for quarters 2 (1430.96) and 3 (6460.23) compared to Quarters 1 (6857.19) and 4 (-1034.01) (Table 3). Overall, the age group 12 -59 months had the highest number of cases 225, 537 (20.3%) while 0 -28 days had the least 30,610 (2.8%). Both malaria death (26.5%) and CFR (44.7 per 100,000) were highest in children 1 to 11 months of age and death was least 63 (26.5%) among in 0 -28 days old (2.5%) (Table 4). Across the years, the data showed a downward trend with a trend line slope of -3287.2 (Figure 2). The highest incidence of malaria cases was in Jaba Local Government Area (47.7%), followed by Sanga (44.5%) and Makarfi (44.1%) and the least in Lere (2.3%), (Figure 3). The seven LGAs with the highest incidence of Malaria are rural LGAs, (Table 5). Zangon Kataf and Igabi LGAs had the highest percentage of complete surveillance report Chikun and Zaria had the least each with 28.3% (Figure 4).

**Table 3:** de-seasonalisation of seasonal variation of reported malaria cases, Kaduna State, 2011-2015

	Quarter			
Year	January- March (Q1)	April - June (Q2)	July - September (Q3)	October - December (Q4)
**2011**	-	-3599.33	2209	11832.67
**2012**	-10239.7	507	16097	-24050
**2013**	6481.67	-3155	4219	8054.33
**2014**	-15473	7832	7879.33	-941.67
**2015**	-9166.33	4359.33	686	-
**Total**	-28397.4	5944	31090.33	-5104.67
**Mean**	-7099.35	1188.8	6218.07	-1276.17
**Residual**	242.16	242.16	242.16	242.16
**βj**	**-6857.19**	**1430.96**	**6460.23**	**-1034.01**

**Table 4:** age distribution of malaria cases, Kaduna State, 2011-2015

Age group	Cases (%)	Deaths (%)	CFR per 1,000
0-28 days	28,885 (2.8)	6 (2.5)	0.09
1-11 months	131,014 (12.7)	63 (26.5)	0.20
12-59 months	209,415 (20.3)	58 (24.4)	0.12
5-9 years	158,867 (15.4)	29 (12.2)	0.08
10-19 years	148,551 (14.4)	26 (10.9)	0.07
20-40 years	153,709 (14.9)	20 (8.4)	0.05
> 40 years	201,163 (19.5)	36(15.1)	0.08
	1,031,603	238	

**Table 5:** incidence of malaria in Kaduna State LGAs, 2015

LGA	Malaria cases	Population size	Incidence (%)	Type of LGA
Lere	9,950	407,286	2.44	Rural
Chikun	17,276	452,901	3.81	Urban
Kaduna North	24,424	439,919	5.55	Urban
Soba	20,656	360,685	5.73	Rural
Kaura	17,139	273,744	6.26	Rural
Zaria	36,800	502,032	7.33	Urban
Sabon gari	29,162	352,815	8.27	Urban
Kauru	25,059	209,088	11.98	Rural
Kachia	36,170	300,426	12.04	Rural
Kaduna South	61,942	494,889	12.52	Urban
Zangonkataf	51,462	389,095	13.23	Rural
Igabi	71,800	529,127	13.57	Urban
Giwa	52,813	352,269	14.99	Rural
Birnin Gwari	48,148	310,375	15.51	Rural
Kudan	31,227	170,943	18.27	Rural
Jema'a	69,059	342,809	20.15	Urban
Kagarko	63,591	296,329	21.46	Rural
Kuban	83,039	346,880	23.94	Rural
Kajuru	36,650	136,354	26.88	Rural
Ikara	75,074	238,505	31.48	Rural
Makarfi	79,241	179,880	44.05	Rural
Sanga	81,717	183,661	44.49	Rural
Jaba	91,088	191,094	47.67	Rural

## Discussion

Overall, the study found a decrease in trend in cases of uncomplicated malaria from 2011 to 2015. Malaria was shown to have higher seasonal variation in second and third quarters. Age group 12 -59 years had the highest number of cases and highest case fatality was among age group 1 -59 months age group. Highest incidence was in Jaba Local Government Area and least in Lere.

It is not surprising that the trend of malaria in Kaduna State has been decreasing over the years. Control measures put in place by the National Malaria Programme include state-wide distribution of LLIN and MIS has showed increased ownership and utilization of these nets. This finding is in concordance with another study done in Kaduna which showed decrease in prevalence of uncomplicated malaria cases by 51% from 2012 -2014 [16]. It may be that the patients presented to the health facilities late or there was inappropriate malaria case management. This may highlight the emphasis on the need for prompt diagnosis and adequate treatment. Seasonal variation was depicted with higher cases recorded in the second and third quarters of the year which is the peak of the rainy season in the State. This is in concordance with other studies that show there is seasonal transmission of malaria in Northern Nigeria [17-19]. The incidence of malaria was higher than 27% reported Ikara, Makarfi, Sanga and Jaba which are rural LGAs. This finding is similar to that reported in MIS 2015 survey which showed that malaria was more common in children from rural areas compared to urban [5].

Another reason that could have accounted for differences in incidence among LGAs is the completeness of surveillance data. The more complete the surveillance data reported is, the more representative it is. Seven out of the twenty-three facilities achieved the standard of 80% reporting rate. This means that there is under reporting of malaria cases in the State. Lere with the lowest incidence of 2.4% had complete surveillance report of only 43.4%. The actual incidence may be higher with complete surveillance data and the differences between LGAs may not be as high as was seen. Igabi and Zangon Kataf both had complete surveillance reports of over 90%, thus the incidence of 13.57% and 13.0% respectively is likely to be representative. Sanga and Jaba with high incidence also have high percentage of complete data 85.0% which means the other LGA may have even higher incidence of they had complete reporting.

The lowest cases and death were recorded among neonates. This may be due to low incidence of congenital malaria in Nigerian newborns [19]. There is some level of immunity at this age conferred by maternal antibodies from the mothers. Moreover, it is possible the neonates have not been exposed to the parasite since they usually sleep under Insecticide TreatedNets. Additionally, our findings revealed that one third of all malaria cases occurred in children under 5 years, this is less than 70% global estimate in the World Malaria Report 2015 [17] but higher than 19% documented in nationwide survey in Côte d’Ivoire [20]. It is however similar to the 42% documented in the Nigeria Demographic and Health Survey 2013 [4]. The study, however, had some limitations. The case definition for malaria was based on presumptive diagnosis and therefore there could have been false positive. The surveillance data reported in this study did not fully represent what could have obtained in the state, as IDSR system only captured data from government-owned health facilities. The completeness of surveillance reports had an influence on incidence and case fatality rates that were generated in the limitation section of the study. In addition while estimating the population size, the death rate was not captured.

## Conclusion

There was a decreased incidence of presumed uncomplicated malaria over the years from 2011 to 2015. Malaria was most common in the second and third quarters. Age group 12-59 months was most affected. Highest incidence was reported in Jaba LGA. The State Malaria Programme should sustain the activities it is presently implementing. The Programme should focus more on the under 5 age group. Additional studies would be useful to identify why Jaba LGA has a high malaria incidence rate and interventions should be targeted appropriately. The DSNOs should be encouraged to report complete data. More research should be done to identify specific LGA differences and specific interventions conducted by the State Malaria Programme.

### What is known about this topic

- Nigeria accounts for up to 29% of malaria disease burden in Africa;
- In Nigeria, 60% of outpatient visits and 30% of hospitalisations among children under 5 is due to malaria;
- Malaria prevalence in Nigerian children under 5 is 27.4%.

### What this study adds

- There was a decrease in trend of uncomplicated malaria cases from 2011 to 2015 in Kaduna State;
- Age group 12 -59 years had the highest number of cases and highest case fatality was among age group 1 -59 months age group;
- There were differences incidence of malaria among Local Government Areas with highest incidence in Jaba Local Government Area and lowest in Lere.

## Competing interests

The authors declare no competing interests.
